# PET/CT imaging of differentiated and medullary thyroid carcinoma using the novel SSTR-targeting peptide [^18^F]SiTATE – first clinical experiences

**DOI:** 10.1007/s00259-024-06944-y

**Published:** 2024-10-15

**Authors:** Sophie C. Kunte, Vera Wenter, Johannes Toms, Simon Lindner, Marcus Unterrainer, Friederike Eilsberger, Klaus Jurkschat, Carmen Wängler, Björn Wängler, Ralf Schirrmacher, Maximilian W. Tiling, Gabriel T. Sheikh, Dirk Mehrens, Matthias Brendel, Johannes Rübenthaler, Christoph J. Auernhammer, Christine Spitzweg, Lena M. Unterrainer, Adrien Holzgreve

**Affiliations:** 1 Department of Nuclear Medicine, LMU University Hospital, LMU Munich, Marchioninistr. 15, 81377 Munich, Germany; 2 DIE RADIOLOGIE, Munich, Germany; 3 Department of Nuclear Medicine, School of Medicine, Philipps University Marburg, Marburg, Germany; 4 Fakultät für Chemie und Chemische Biologie, Technische Universität Dortmund, Dortmund, Germany; 5 Biomedical Chemistry, Clinic of Radiology and Nuclear Medicine, Medical Faculty Mannheim of Heidelberg University, Mannheim, Germany; 6 Research Campus M²OLIE, Medical Faculty Mannheim of Heidelberg University, Mannheim, Germany; 7 Medical Faculty Mannheim of Heidelberg University, Molecular Imaging and Radiochemistry, Clinic of Radiology and Nuclear Medicine, Mannheim, Germany; 8 Department of Oncology, Division of Oncological Imaging, University of Alberta, Edmonton, AB Canada; 9 Department of Radiology, LMU University Hospital, LMU Munich, Munich, Germany; 10 DZNE – German Center for Neurodegenerative Diseases, Munich, Germany; 11 Munich Cluster for Systems Neurology (SyNergy), University of Munich, Munich, Germany; 12 German Cancer Consortium (DKTK), Partner Site Munich, a Partnership Between DKFZ and Ludwig-Maximilians-Universität München (LMU), Munich, Germany; 13 Department of Internal Medicine IV, LMU University Hospital, LMU Munich, Munich, Germany; 14 Ahmanson Translational Theranostics Division, David Geffen School of Medicine at UCLA, Los Angeles, CA USA; 15 Bayerisches Zentrum für Krebsforschung (BZKF), Partner Site Munich, Munich, Germany

**Keywords:** (4–6): Medullary thyroid carcinoma (MTC), Differentiated thyroid carcinoma (DTC), Follicular thyroid carcinoma (FTC), Papillary thyroid carcinoma (PTC), Somatostatin receptor (SSTR), Serum tumor marker

## Abstract

**Purpose:**

The novel ^18^F-labeled somatostatin receptor (SSTR)-directed radiotracer [^18^F]SiTATE demonstrated promising results for the imaging of various SSTR-expressing tumor types. Although thyroid carcinomas (TC) express SSTR, data on [^18^F]SiTATE PET/CT imaging in TC are lacking. This study explores the use of [^18^F]SiTATE PET/CT in a patient cohort with histologically proven TC.

**Methods:**

As part of a prospective observational study at a single tertiary cancer center, 21 patients with TC (10 medullary (MTC) and 11 differentiated (DTC)) who underwent at least one [^18^F]SiTATE PET/CT were included (37 scans in total). Mean SUV_max_ and SUV_mean_ of tumoral lesions, mean total-tumor-volume (TTV), and whole-body (WB)-SUV_max_ and WB-SUV_mean_ on PET with their standard deviations (SDs) were determined. PET parameters were correlated to clinical parameters including tumor marker levels (thyroglobulin for DTC, calcitonin for MTC).

**Results:**

89 lesions were included in the analysis. Metastases were localized in the bone, lymph nodes, lung, soft tissue, and thyroid bed. Osseous (31 lesions; SUV_max_ 8.6 ± 8.0; SUV_mean_ 5.8 ± 5.4) and nodal (37 lesions; SUV_max_ 8.7 ± 7.8; SUV_mean_ 5.7 ± 5.4) metastases showed the highest uptake. The MTC disease burden on PET significantly correlated with the calcitonin tumor marker level (e.g., TTV: *r* = 0.771, r^2^ = 0.594, *p* = 0.002). For DTC, no such correlation was present.

**Conclusion:**

Our data demonstrate high feasibility of [^18^F]SiTATE PET/CT in a small cohort of patients with MTC and DTC. The use of [^18^F]SiTATE may overcome logistical disadvantages of ^68^Ga-based tracers and facilitate SSTR-targeted PET/CT imaging of thyroid carcinoma.

## Background

Thyroid cancer (TC) is one of the most common cancers in women and shows a globally increasing incidence [1]. The age-standardized incidence rate was determined to be 10.1 per 100,000 women and 3.1 per 100,000 men [2]. Even if TC accounts for only 2.2% of all new cancer cases, it is suspected that TC will replace colorectal cancer as the fourth leading cancer entity by 2030 [3, 4]. The classification of TC relies on several mostly pathologic features including the cell of origin [5]. The most frequent differentiated carcinomas are traditionally grouped as follicular (FTC), and papillary (PTC) thyroid carcinomas. Oncocytic carcinoma of the thyroid (formerly known as “Hürthle cell” carcinoma) constitutes another group of follicular-cell derived malignant neoplasm. Medullary thyroid carcinoma (MTC) is a distinct, less frequent tumor class derived from thyroid C cells [5]. The most specific imaging for DTC after thyroidectomy is whole-body ^131^I-scintigraphy as long as the tumor is capable of accumulating radioiodine due to functional expression of the sodium iodide symporter (NIS) [6]. At advanced disease stages, in particular in radioiodine-refractory tumors, the interdisciplinary management of DTC requires additional imaging modalities such as 2-[^18^F]fluoro-2-deoxy-D-glucose ([^18^F]FDG) positron emission tomography / computed tomography (PET/CT) and magnetic resonance imaging [7]. MTC derives from thyroidal C cells and is therefore a priori not susceptible to radioiodine-based imaging. Several PET tracers are proposed for staging and recurrence detection of MTC, including [^18^F]FDG, 6-[^18^F]fluoro-L-DOPA ([^18^F]FDOPA) and somatostatin receptor (SSTR) radioligands [8].

In the last decade, ^68^Ga-labeled SSTR radioligands such as [^68^Ga]Ga-DOTA-TATE, [^68^Ga]Ga-DOTA-TOC and [^68^Ga]Ga-DOTA-NOC have been increasingly evaluated for the imaging of TC, as SSTRs are expressed on the cell surface of TC at various levels [9, 10] and represent an attractive theranostic target. For both DTC and MTC, studies demonstrated a high correlation in the detection rate of metastatic sites between SSTR-PET and [^18^F]FDG PET/CT [11, 12]. Beyond merely diagnostic purposes, the use of SSTR-PET might also help to select patients that could benefit from a SSTR-directed peptide receptor radionuclide therapy (PRRT) in a theranostic approach [13]. The increasing availability of PRRT and its growing application in entities other than gastro-entero-pancreatic neuroendocrine tumors (GEP-NETs) may further drive the use of SSTR-PET/CT imaging in TC [14, 15].

The radiotracer [^18^F]SiTATE is a novel SSTR-targeting peptide that has shown promising results in patients with various neuroendocrine tumors, overcoming logistical disadvantage of the established ^68^Ga-based SSTR radioligands [16–18]. This study aimed to investigate the feasibility of [^18^F]SiTATE PET/CT in a patient cohort diagnosed with histologically proven differentiated thyroid carcinoma (DTC) or medullary thyroid carcinoma (MTC). We compared PET-derived results with the CT-based metastatic assessment and the serum level of the respective tumor markers.

## Materials and methods

### Study design and patients

As part of a prospective observational study at a tertiary cancer center, we included patients with histologically proven thyroid cancer who were referred by their treating oncologists for [^18^F]SiTATE PET/CT imaging in clinical routine. All patients gave written consent to undergo [^18^F]SiTATE PET/CT. The analysis was performed in compliance with the principles of the Declaration of Helsinki and was approved by the institutional ethics committee of the Ludwig-Maximilians-University (LMU) of Munich (IRB #21–0102). General patient characteristics included age and sex. Clinical and tumor-specific characteristics included histological subtype, genetic mutations, oncologic treatments, and serum tumor marker levels. PET-derived results were compared with the CT-based metastatic assessment and clinical parameters.

## Radiopharmaceutical preparation and imaging protocol

The SiTATE precursor was obtained from ABX Advanced Biochemical Compounds (Radeberg, Germany) and radiosynthesis of [^18^F]SiTATE was performed at the Department of Nuclear Medicine at LMU Munich, Germany, conform to clinical good manufacturing practice as previously described [19–21]. Quality control measurements were in accordance with local product release criteria and patient application was conducted in accordance with the German Medicinal Products Act § 13(2b). A weight-adapted [^18^F]SiTATE dose was injected intravenously at a median activity of 215.5 MBq (range, 113–353 MBq). All [^18^F]SiTATE PET/CT scans were acquired at the Department of Nuclear Medicine at LMU Munich using a Siemens Biograph mCT flow (Siemens Healthineers, Erlangen, Germany). Image acquisition started at a median of 90 min (range 45–138 min) after tracer injection and recorded for 20 min [16]. If no medical contraindication was given, patients received furosemide as a premedication for radiation protection and 1.5 mL of iopromide (Ultravist-300, Bayer Healthcare, Leverkusen, Germany) per kg body weight to obtain contrast-enhanced, diagnostic CT scans in portal-venous phase. PET images were reconstructed iteratively using TrueX (3 iterations and 21 subsets, 3D Gauss post-filter of 4-mm full width half maximum). Slice thickness on CT was 0.3 cm.

## Image analysis

A dedicated software package was used for PET analysis (Hermes Hybrid Viewer, Affinity 1.1.4; Hermes Medical Solutions, Stockholm, Sweden). Tumoral [^18^F]SiTATE uptake was determined by SUV_max_ and SUV_mean_ (± standard deviation, SD) of the single lesions using a 50%-isocontour volume-of-interest. Total-tumor-volume (TTV), whole-body (WB)-SUV_max_, WB-SUV_mean_, and respective SD were evaluated using a SUV threshold of 4.0. In some patients, several [^18^F]SiTATE PET/CTs were available and the course of the disease was determined comparing the respective PET results according to PERCIST 1.0 [22].

The evaluation of CT datasets was performed on a dedicated software (mint lesion™, version 3.8.6, Mint Medical GmbH, Dossenheim, Germany). Target and non-target lesions according to RECIST 1.1 (target lesions: ≥1.5 cm short-axis diameter) were defined on the first [^18^F]SiTATE PET/CT (baseline) and at follow-up, if available [23]. Lesions were manually measured.

Imaging-derived parameters, e.g. SUV_max_ and SUV_mean,_ were correlated with the respective serum tumor marker levels. Additionally, PET-derived parameters were compared between the histological tumor entities.

### Statistical analysis

Data analysis was performed using Microsoft Excel (Excel 2019, Microsoft, Redmond, WA, USA) and GraphPad Prism (Version 9.5.0 (730), GraphPad Software, Boston, Massachusetts USA). Descriptive statistics are displayed as median (range) or mean ± standard deviation (SD). Shapiro-Wilk normality test was performed. Correlation analysis using Spearman or Pearson test was conducted. Group differences were analyzed using a t-test or Mann-Whitney-test. A two-tailed p-value < 0.05 was considered statistically significant.

## Results

### Patient characteristics

21 patients with TC were included (mean age 62.2 ± 13.9 years; 13 female / 8 male). Ten patients were diagnosed with MTC (47.6%), nine with FTC (42.9%; thereof one patient with oncocytic carcinoma) and two with PTC (9.5%). Twelve patients underwent one single [^18^F]SiTATE PET/CT scan (57.1%), four patients received two scans (19.0%), three patients received three scans (14.3%) and two patients obtained four scans (9.5%). The indications for PET imaging were initial staging, follow-up, and PRRT eligibility and are listed in Table 1. Prior to PET/CT, 19 patients had undergone thyroidectomy (90.5%), nine patients radioactive iodine therapy (42.9%), four patients received [^177^Lu]Lu-DOTA-TATE therapy (19.0%), and five patients received radiation therapy (23.8%). One MTC patient received vandetanib, and another one octreotide and selective internal radiation therapy (SIRT) (6.7%, respectively) prior to [^18^F]SiTATE PET/CT depending on the treatment options available at that time. Patient characteristics including treatments are given in Table 1.

**Table 1:** Patient characteristics

Pat.	Age	Sex	Histology	Genetic mutations	Prior therapy					Indication (1: baseline, 2: follow up, 3 PRRT eligibility)
					Thyroidectomy	Radioactive iodine	Radiotherapy	[^177^Lu]Lu-DOTA-TATE	Others	
1	75	f	FTC		X	X		4 cycles		2, 3
2	71	m	FTC		X	X		8 cycles		2, 3
3	76	f	FTC		X	X				2, 3
4	67	f	FTC		X	X				2
5	74	m	FTC *			X	X	3 cycles		2, 3
6	71	f	FTC		X	X	X			2
7	88	m	FTC	KRAS						2
8	80	m	FTC		X	X	X			3
9	71	f	FTC		X	X	X	4 cycles		2
10	64	f	MTC	HRAS	X					2
11	47	f	MTC	RET	X					2
12	73	m	MTC		X					2
13	52	m	MTC	RET	X					2
14	58	m	MTC		X					1
15	57	f	MTC		X					2
16	40	m	MTC		X		X		Vandetanib	2
17	53	f	MTC		X					2
18	52	f	MTC	RET	X				SIRT, Octreotide^‡^	2
19	60	f	MTC	KRAS, TP53	X		X			2
20	44	f	PTC		X	X				1
21	34	f	PTC		X					2

F: female, m: male, FTC: follicular thyroid carcinoma, MTC: medullary thyroid carcinoma, PTC: papillary thyroid carcinoma; thyroid-stimulating hormone suppressive therapy in DTC not listed, X: patient received the respective treatment prior to PET/CT scan. * Now classified as Oncocytic carcinoma of the thyroid (formerly Hürthle cell carcinoma) ^‡^Based on the treatment options available at that time; following genetic testing, treatment with a selective RET inhibitor was offered to the patient

15/21 patients received prior PET imaging using other tracers than SSTR radioligands (*n* = 12 [^18^F]FDG; *n* = 3 [^18^F]FDOPA), however only in two cases the PET scan occurred at a time interval < 3 months to the [^18^F]SiTATE PET/CT scan. Due to the long time span between [^18^F]SiTATE PET/CT and the further PET scan as part of clinical routine in all other cases (13/15; 86.7%), a head-to-head comparison of uptake characteristics of different tracer types was not feasible in this analysis.

## [^18^F]SiTATE uptake characteristics in PET

**Fig. 1:**
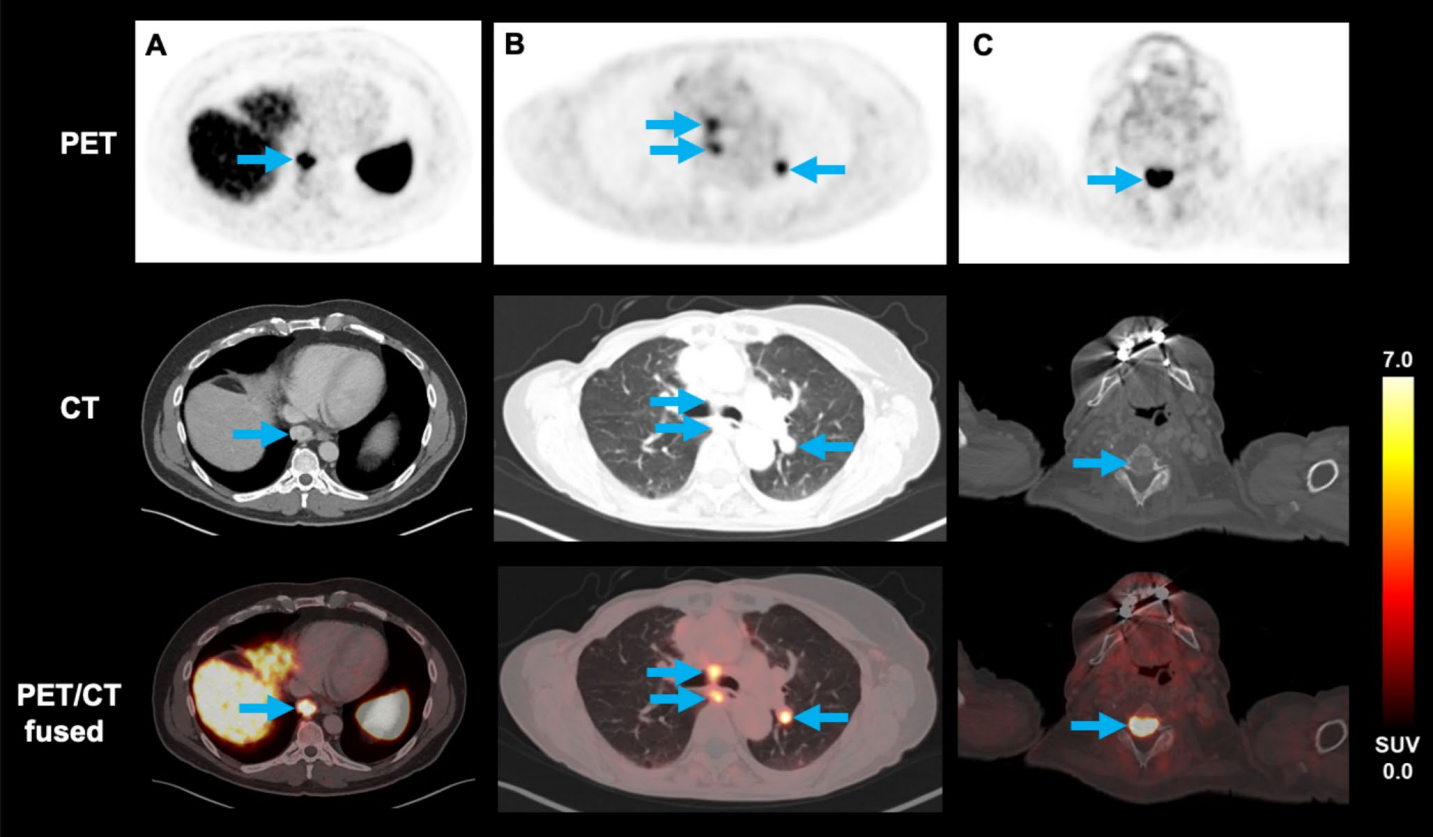
PET/CT images using the novel radiotracer [^18^F]SiTATE in patients with a mediastinal metastasis of MTC **(A)**, with nodal and pulmonary metastases of FTC **(B)** and an osseous metastasis of FTC **(C)**

Altogether, 89 metastatic lesions were included. 5/21 patients had no evidence of metastatic sites on their first [^18^F]SiTATE PET/CT scan: Three patients with MTC and one patient with FTC and PTC respectively showed no suspicious [^18^F]SiTATE uptake, which was in line with no target lesions on CT. Analysis of osseous (31 lesions; SUV_max_ 8.6 ± 8.0; SUV_mean_ 5.8 ± 5.4) and nodal (37 lesions; SUV_max_ 8.7 ± 7.8; SUV_mean_ 5.7 ± 5.4) metastases showed the highest uptake. Metastases were also localized in the lung (17 lesions; SUV_max_ 4.5 ± 1.9; SUV_mean_ 3.5 ± 1.7) and in the soft tissue (gluteal, 1 lesion; SUV_max_ 4.1; SUV_mean_ 2.4). The SUV_max_ and SUV_mean_ of osseous (*p* = 0.015 and *p* = 0.003) as well as nodal (*p* < 0.001 and *p* = 0.002) lesions was significantly higher in DTC compared to MTC. There was no significant group difference for the uptake of pulmonary lesions (SUV_max_, *p* = 0.182; SUV_mean_, *p* = 0.077) (Table 2). Exemplary [^18^F]SiTATE PET/CT images of TC patients with mediastinal, pulmonary, and osseous metastases are shown in Fig. 1.

**Table 2:** [^18^F]SiTATE uptake characteristics in PET

	Overall		DTC		MTC		*p*-value (DTC vs. MTC)	
	SUV_max_	SUV_mean_	SUV_max_	SUV_mean_	SUV_max_	SUV_mean_	SUV_max_	SUV_mean_
**Osseous**	8.6 ± 8.0	5.8 ± 5.4	15.0 ± 11.9	10.3 ± 7.8	5.6 ± 1.3	3.7 ± 0.9	0.015	0.003
**Pulmonary**	4.5 ± 1.9	3.5 ± 1.7	5.0 ± 2.0	4.0 ± 1.9	3.7 ± 1.5	2.5 ± 0.8	0.182	0.077
**Nodal**	8.7 ± 7.8	5.7 ± 5.4	13.1 ± 11.0	8.5 ± 7.7	6.0 ± 2.9	4.0 ± 2.0	< 0.001	0.002
**Soft tissue**	4.1	2.4	n.a.	n.a.	4.1	2.4	n.a.	n.a.
**Thyroid**	12.0 ± 11.8	7.5 ± 6.9	16.0 ± 13.5	10.0 ± 7.5	3.9	2.5	n.a.	n.a.

DTC differentiated thyroid carcinoma; MTC medullary thyroid carcinoma; n.a. not applicable

One patient did not undergo thyroidectomy prior to imaging and in two patients, a local recurrence was present; the local tumoral uptake was high in all three cases (SUV_max_ 12.0 ± 11.8; SUV_mean_ 7.5 ± 6.9; see Table 2).

In patients with DTC, mean total tumor volume (TTV) was 539.2 ± 1059.9 mL, WB-SUV_max_ 14.6 ± 14.7, and WB-SUV_mean_ 5.3 ± 3.0. In patients with MTC, mean WB-TTV was 77.0 ± 173.1 mL, WB-SUV_max_ 5.5 ± 4.2 and WB-SUV_mean_ 3.6 ± 2.4.

## Correlation of PET-derived parameters and serum tumor marker levels

**Fig. 2:**
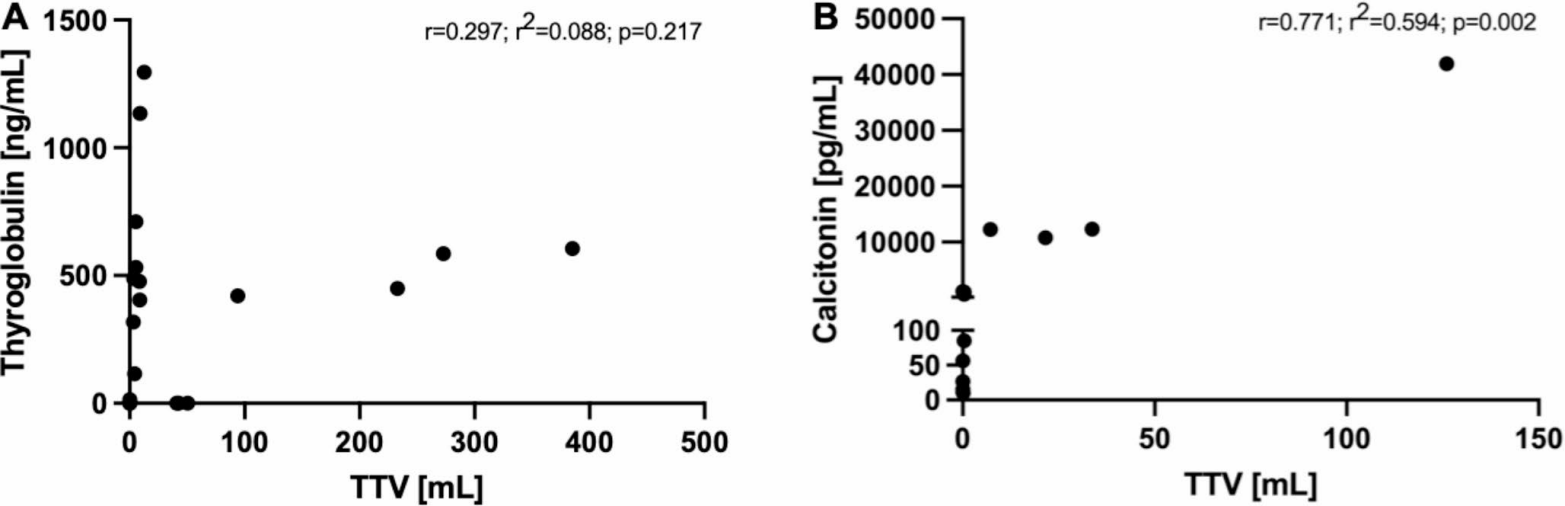
**(A)** No significant correlation of the total tumor volume and the thyroglobulin level (*r* = 0.297; r^2^ = 0.088; *p* = 0.217) in patients with DTC. **(B)** Significant correlation of the total tumor volume and the calcitonin level (*r* = 0.771; r^2^ = 0.594; *p* = 0.002) in patients with MTC. DTC differentiated thyroid carcinoma; MTC medullary thyroid carcinoma; TTV total tumor volume

**Fig. 3:**
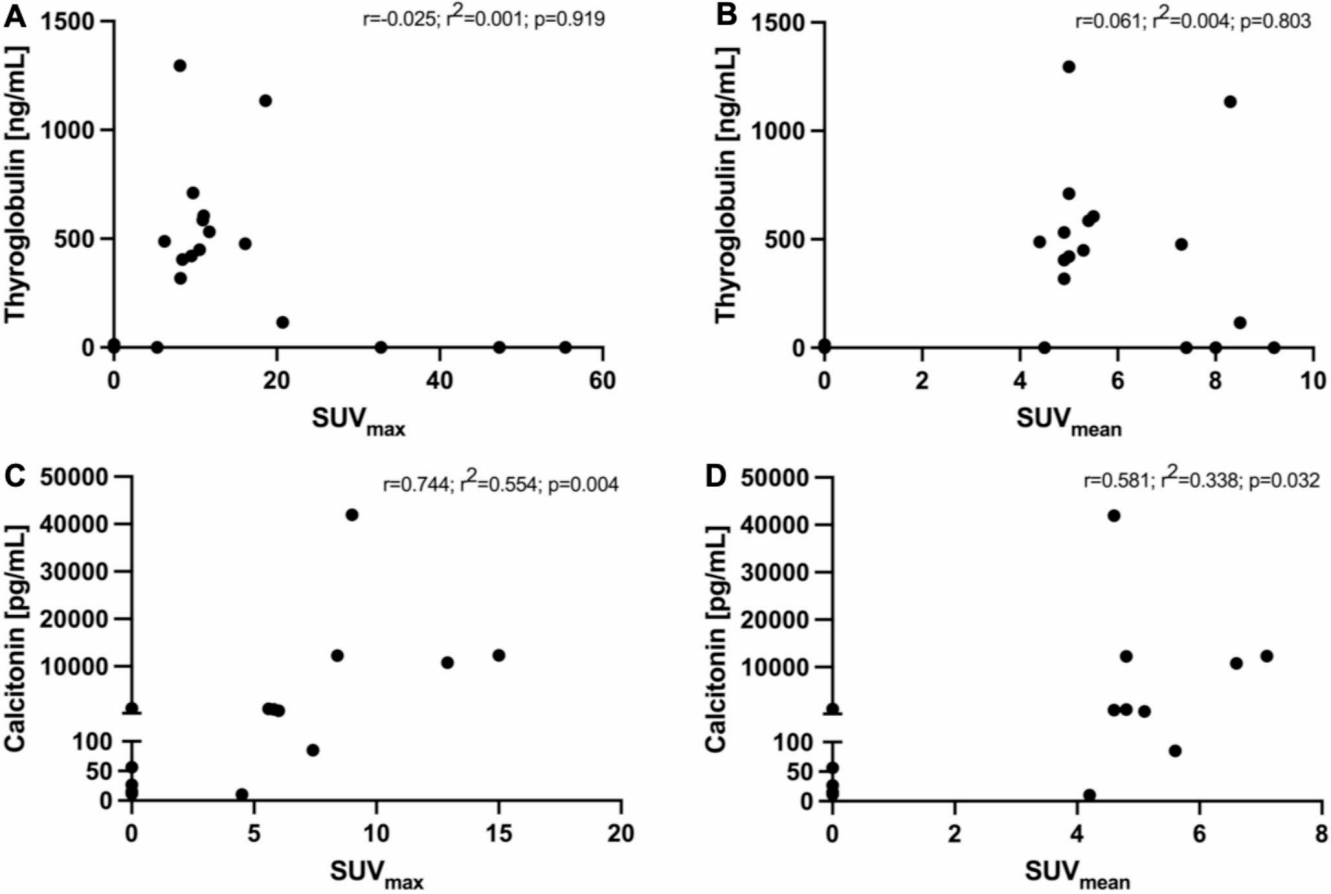
No significant correlation of the SUV_max_ (*r*=-0.025; r^2^ = 0.001; *p* = 0.919) **(A)** or SUV_mean_ (*r* = 0.061; r^2^ = 0.004; *p* = 0.803) **(B)** and the thyroglobulin level in patients with DTC. Significant correlation of the SUV_max_ (*r* = 0.744; r^2^ = 0.554; *p* = 0.004) **(C)** or SUV_mean_ (*r* = 0.581; r^2^ = 0.338; *p* = 0.032) **(D)** and the calcitonin level in patients with MTC. DTC differentiated thyroid carcinoma; MTC medullary thyroid carcinoma

There was no significant correlation of the serum thyroglobulin level with TTV (*r* = 0.297, r^2^ = 0.088, *p* = 0.217), WB-SUV_max_ (*r*=-0.025, r^2^ = 0.001, *p* = 0.919) and WB-SUV_mean_ (*r* = 0.061, r^2^ = 0.004, *p* = 0.803) in patients with DTC (Figs. 2A and 3A-B). In patients with MTC, the serum calcitonin level strongly correlated with the PET parameters TTV (*r* = 0.771, r^2^ = 0.594, *p* = 0.002), WB-SUV_max_ (*r* = 0.744, r^2^ = 0.554, *p* = 0.004) and WB-SUV_mean_ (*r* = 0.581, r^2^ = 0.338, *p* = 0.032) (Figs. 2B and 3C-D).

**Fig. 4:**
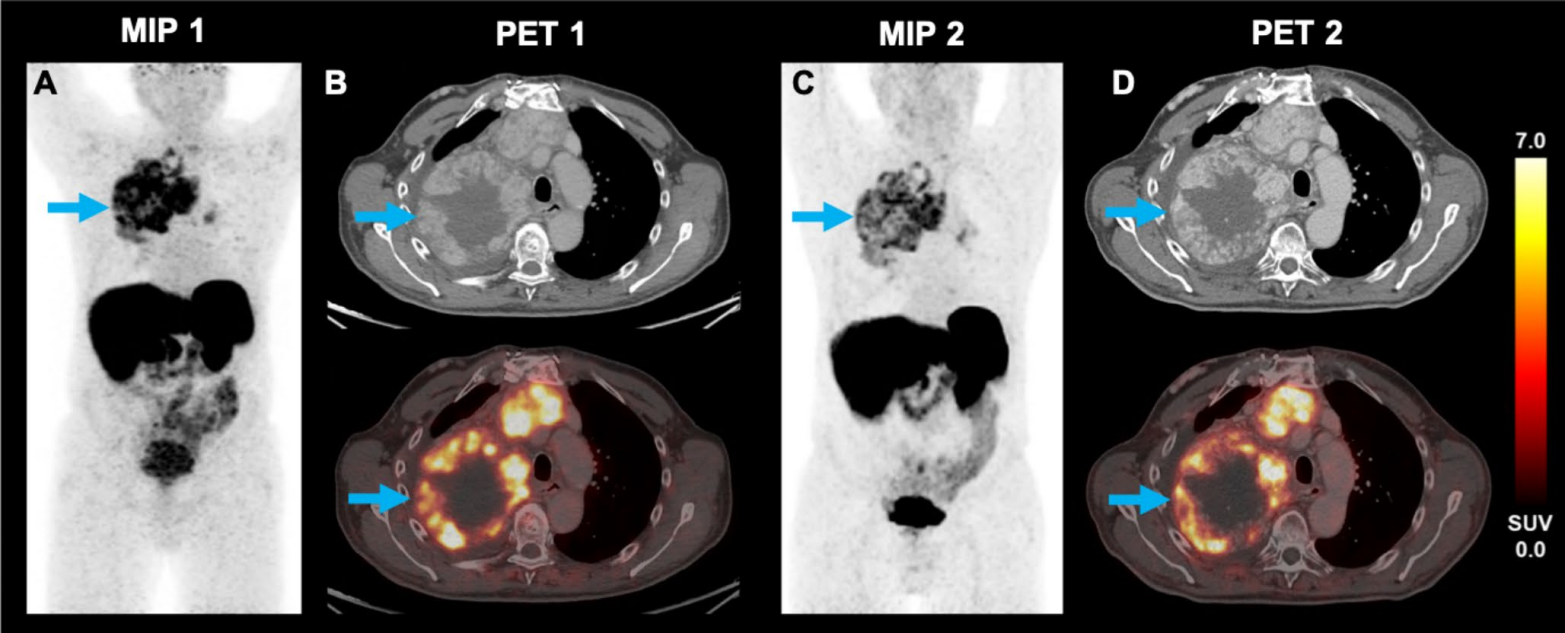
A 74-year-old male patient diagnosed with oncocytic carcinoma of the thyroid (formerly Hürthle cell variant FTC), who underwent iodine radioactive therapy and radiotherapy, was referred to our department due to a high thyroglobulin level of 606 ng/mL. The maximum intensity projection (MIP) and selected transversal levels of a mediastinal tumor bulk (blue arrow) of the baseline and follow-up PET are shown. The baseline [^18^F]SiTATE PET/CT (**A**-**B**) revealed a TTV of 385 mL. Metastases were located mediastinal, bipulmonary and hilar. After two cycles of [^177^Lu]Lu-DOTA-TATE therapy the thyroglobulin level was 273 ng/mL and [^18^F]SiTATE PET/CT showed a TTV of 273 mL (**C**-**D**), suggestive for partial response

**Fig. 5:**
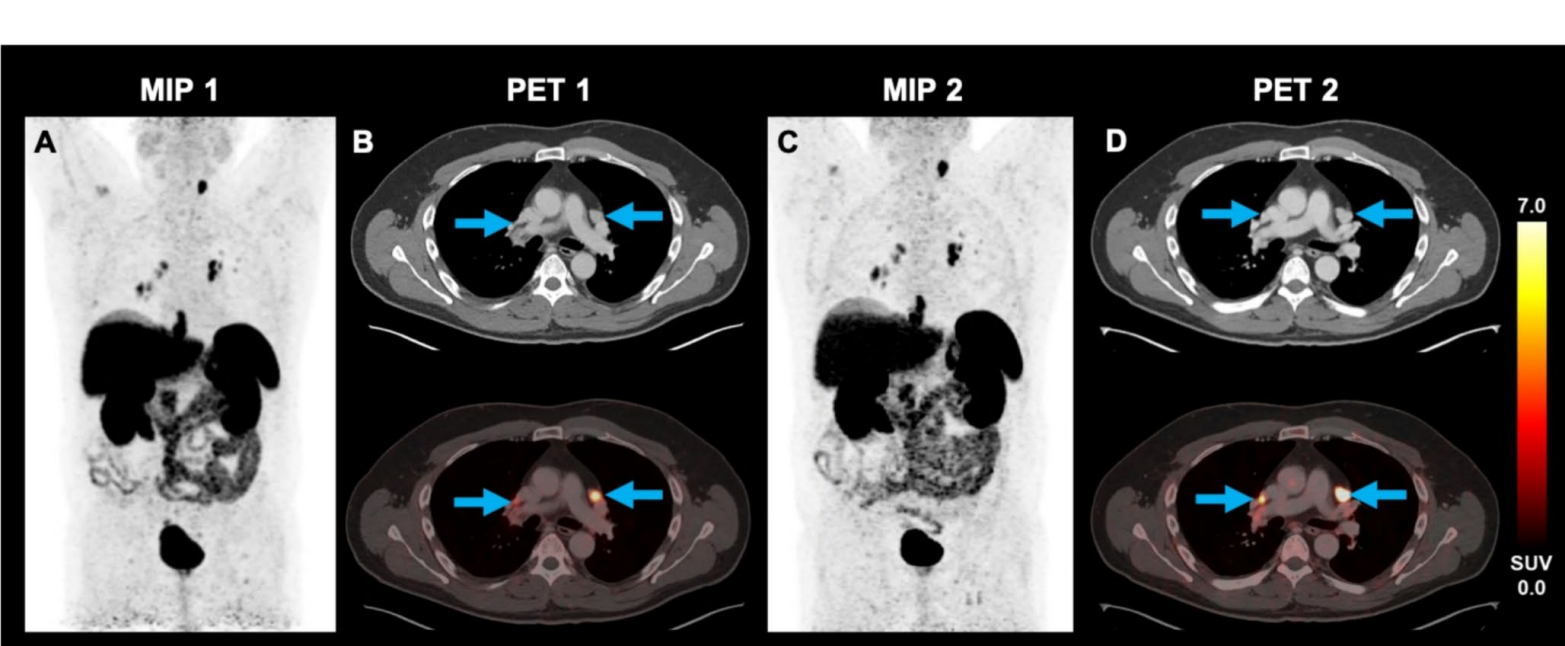
A 52-year-old male patient diagnosed with medullary thyroid carcinoma underwent thyroidectomy and was referred to our department due to a calcitonin level of 10,797 pg/mL. The maximum intensity projection (MIP) and selected transversal levels of hilar metastases (blue arrows) of the baseline and follow-up PET are shown. The baseline [^18^F]SiTATE PET/CT showed a TTV of 21.5 mL (**A**-**B**). Metastases were located cervical, mediastinal, bipulmonary and hilar. After 11 months of a watchful waiting strategy both the calcitonin level (12,329 pg/mL) and the TTV (33.7 mL) on the follow-up [^18^F]SiTATE PET/CT increased (**C**-**D**)

The cohort included one patient diagnosed with oncocytic carcinoma (formerly known as Hürthle cell variant FTC). Figure 4 shows exemplary [^18^F]SiTATE PET/CT images of this patient, who was referred from an external hospital after ineffective radiation therapy of a mediastinal tumor. As the patient showed highly increased SSTR expression on PET in all solid tumor parts, PRRT seemed promising in this case and was offered as a personalized treatment approach. After two cycles of [^177^Lu]Lu-DOTA-TATE therapy, tumor growth was halted (TTV decreased from 385 mL to 273 mL) and the thyroglobulin tumor marker level decreased from 606 ng/ml to 273 ng/mL. Figure 5 shows the [^18^F]SiTATE PET/CT images of a 52-year-old patient with metastatic MTC who was referred due to a rising calcitonin level (10,797 pg/mL). The patient had previously received [^18^F]FDOPA PET/CTs for lung nodules that slowly progressed in size over three years, without showing increased tracer uptake. An [^18^F]FDG PET/CT scan one year later showed increased uptake in thoracic lymph nodes but not in the lung nodules. Further follow-up scans included [^18^F]FDOPA PET (negative) and [^68^Ga]Ga-DOTA-TOC PET/CT. The latter showed strong SSTR radioligand uptake both in cervical and thoracic lymph nodes, and in the lung nodules. As the WB-TTV was low (21.5 mL), a watchful waiting strategy was decided. After additional 11 months, the WB-TTV (33.7 mL) on [^18^F]SiTATE PET/CT and the calcitonin level (12,329 pg/mL) showed a concurrent increase.

### Comparison of [^18^F]SiTATE and [^68^Ga]Ga-DOTA-TATE uptake in PET

Out of 21 patients, 8 patients received a [^68^Ga]Ga-DOTA-TATE PET/CT imaging at an average of 7.6 ± 2.8 months before the [^18^F]SiTATE PET/CT. 4 patients were diagnosed with FTC, 3 with MTC and 1 with PTC. Due to the long time span between the two PET scans as part of clinical routine in the respective cases, the comparison of the modalities serves only as an approximate orientation with regard to the localization of the metastases.

**Fig. 6:**
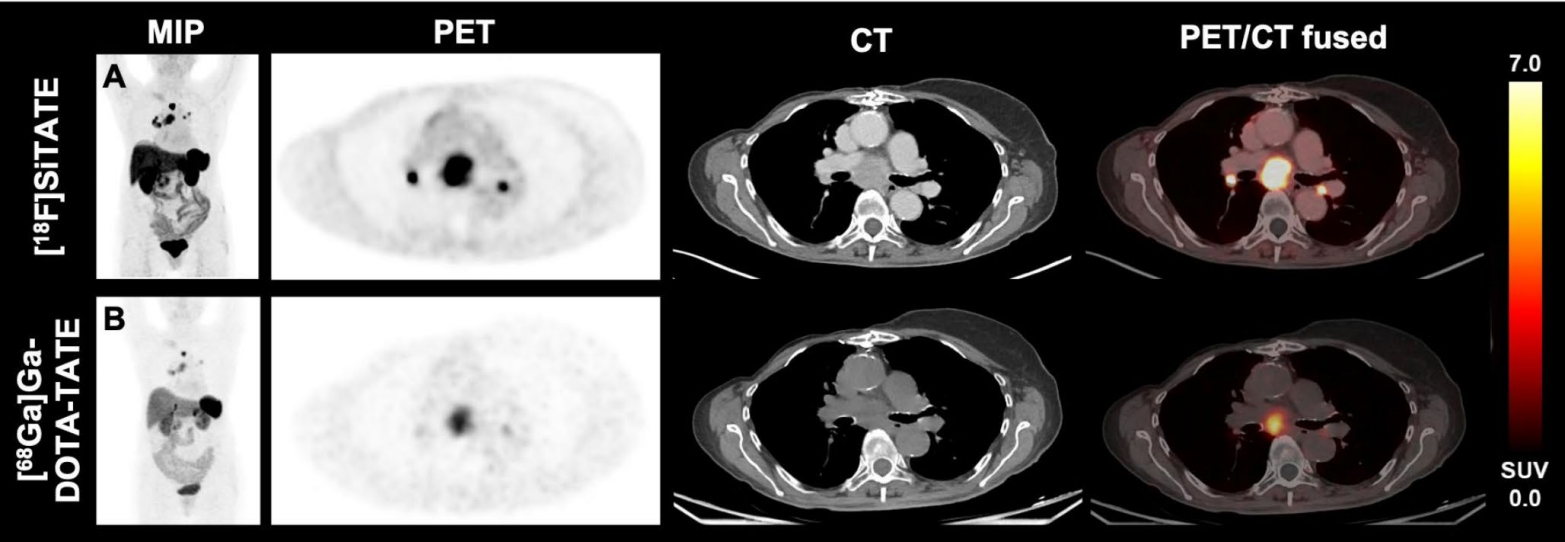
PET/CT imaging of a 75-year-old female patient diagnosed with FTC using the tracers [^18^F]SiTATE **(A)** and [^68^Ga]Ga-DOTA-TATE **(B)**. The maximum intensity projection (MIP) and selected transversal PET and CT levels of the baseline and follow-up imaging are shown. The figure demonstrates mediastinal and hilar tumor manifestations. During the interval of 7 months the tumor volume increased by 29.2% and SUV_mean_ increased by 60.0%, suspicious of tumor progression

Figure 6 illustrates PET images, featuring the maximum intensity projection (MIP) and specific tumor lesions of a 75-year-old female patient diagnosed with follicular thyroid carcinoma (FTC), comparing [^18^F]SiTATE (A) and [^68^Ga]Ga-DOTA-TATE (B). The localization of metastases showed comparable results in both imaging modalities. However, over a 7-month interval, the tumor volume increased by 29.2% and the SUV_mean_ by 60.0%, indicating progressive disease on PET, consistent with progressive disease observed on CT.

**Fig. 7:**
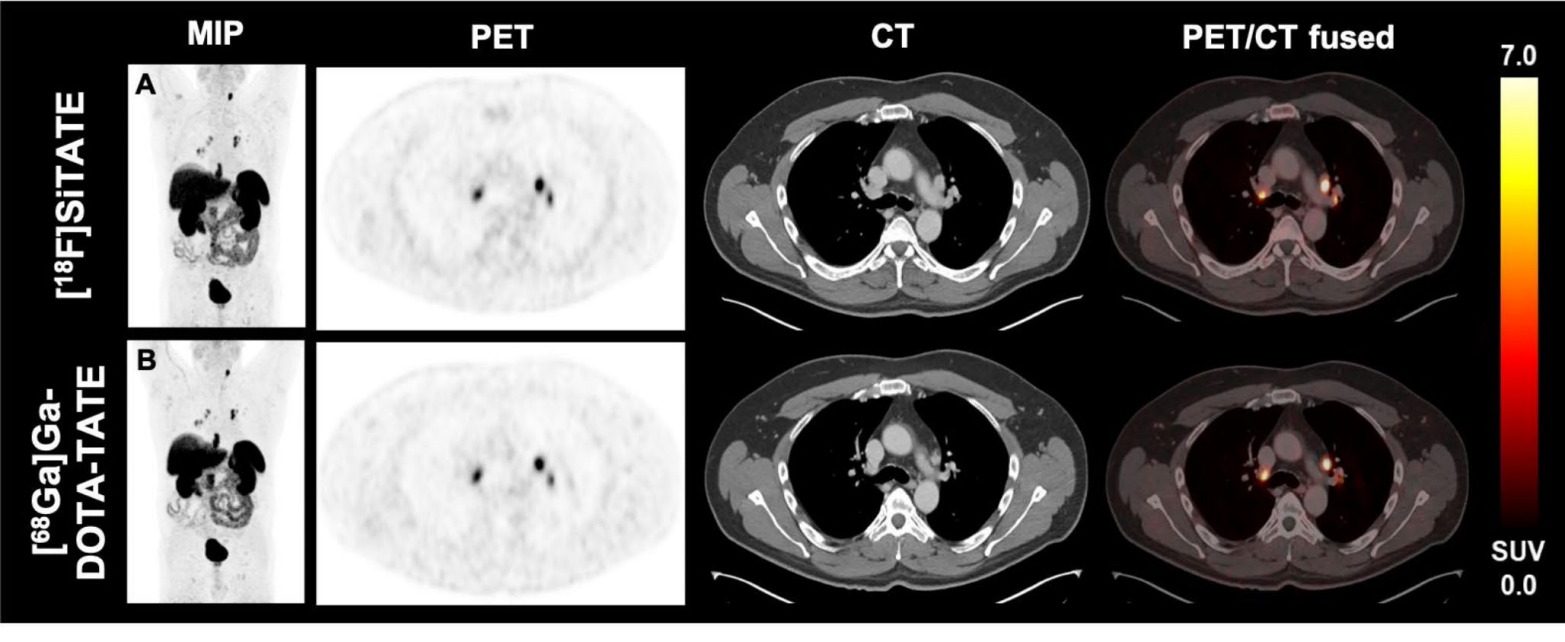
PET/CT imaging of a 52-year-old male patient diagnosed with MTC (patient 13) using the tracers [^18^F]SiTATE **(A)** and [^68^Ga]Ga-DOTA-TATE **(B)**. The maximum intensity projection (MIP) and selected transversal levels of the baseline and follow-up PET are shown. The figure demonstrates hilar tumor manifestations. During the interval of 6 months the tumor volume increased by 14.6% and SUV_mean_ decreased by 5.7%, indicating stable disease

Figure 7 demonstrates PET images including the MIP and selected tumor lesions of a 52-year-old male patient with MTC comparing [^68^Ga]Ga-DOTA-TATE and [^18^F]SiTATE. During the interval of 6 months, the TTV increased by 14.6% and the SUV_mean_ decreased by 5.7%, thus defined as stable disease on PET in line with stable disease according to CT-based criteria.

## Discussion

Our data in a small cohort of patients diagnosed with DTC and MTC demonstrate high feasibility of [^18^F]SiTATE PET/CT for thyroid cancer imaging. In all cases, increased [^18^F]SiTATE uptake on PET was noted at metastatic sites as localized on CT. These preliminary exploratory data suggest that [^18^F]SiTATE could be a valuable new tracer for assessing thyroid cancer patients, offering advantages over ^68^Ga-labeled SSTR radioligands.

^68^Ga-labeled SSTR radioligands are widely used in the imaging of different tumor entities, such as GEP-NET or meningioma [24, 25]. However, the use of ^68^Ga-labeled tracers comes with disadvantages. The production of ^68^Ga-labeled tracers requires expensive ^68^Ge/^68^Ga-generators and ^68^Ga has a short half-life of 68 minutes, thus the production of only small amounts is possible. [^18^F]SiTATE addresses these drawbacks by being producible through a in principle less expensive cyclotron-based procedure, yielding higher activities per synthesis and enabling imaging of more patients per batch. Furthermore, ^18^F has a longer half-time of 110 min and the spatial resolution of ^18^F is higher in comparison to ^68^Ga due to a lower positron energy (mean positron energy ^18^F: 0.25 MeV, 0.6 mm vs. ^68^Ga: 0.83 MeV, 3.5 mm) [17, 20, 21]. Overall, ^18^F-labeled SSTR radioligands are of great interest and their clinical application is on the rise [26, 27]. Although [^18^F]SiTATE PET/CT has proven high feasibility in several entities [17, 28], data on [^18^F]SiTATE PET/CT imaging in thyroid cancer have not been available until now.

MTC is an epithelial neuroendocrine neoplasm that may inherently overexpress SSTR. Therefore, SSTR-targeted PET is a suitable imaging procedure for MTC, although it has to be noted that not all MTC cases are SSTR-positive [8]. SSTR-targeted PET imaging is listed in nuclear medicine guidelines for MTC imaging e.g. to detect and localize recurrent disease in case of postoperatively increased serum levels of MTC markers with corresponding negative or inconclusive morphologic imaging [8]. An overview of the role of SSTR imaging in diagnostic work-up of MTC patients as stated in current guidelines is given in Table 3. In our analysis, total tumor volume and tracer uptake intensity as depicted on PET significantly correlated with serum calcitonin tumor marker level, supporting that [^18^F]SiTATE might be able to evaluate changes in disease burden, e.g. in cases of unclear tumor marker elevation. In this sense, the tumor marker level and its doubling time could serve as yardsticks to inform SSTR-based imaging. Other PET tracers established for MTC imaging include [^18^F]FDG and [^18^F]FDOPA. A previously published head-to-head comparison of PET imaging with [^18^F]FDG, [^18^F]FDOPA, and a ^68^Ga-labeled SSTR radioligand suggested superior diagnostic performance of [^18^F]FDOPA resulting in a significantly higher proportion of change in patient management [29]. [^18^F]FDOPA PET/CT may be the most accurate method to assess disease extent in patients with recurrent MTC and usually serves as the first-line procedure to restage MTC [8]. Yet, regarding tumor staging the different available imaging modalities for MTC rather seem to complement each other and other tracers may be superior depending on the specific clinical situation [8]. Thus, if the tumor marker level seems “unexpected” compared to the staging information obtained from other diagnostic imaging modalities, the acquisition of a supplemental SSTR-targeted PET scan should be considered. Here, the unique selling point of SSTR-based radioligands such as [^18^F]SiTATE is the applicability for patient selection in a theranostic setting, as it is the only option to assess patients eligible for PRRT when other therapy options are lacking [30]. While the approach has been proven to be safe in patient series treated so far, high class evidence for treatment efficacy of PRRT in MTC is still missing [30].

**Table 3:** Overview of the role of SSTR imaging in diagnostic work-up of thyroid cancer patients as stated in current guidelines

SNMMI Procedure Standard/EANM Practice Guideline for Nuclear Medicine Evaluation and Therapy of Differentiated Thyroid Cancer [39] SSTR imaging as tertiary tier in order to - detect aggressive histologic variants of DTC (independently of glucose transport overexpression) - assess complementary information in FDG-positive patients with poorly differentiated or oxyphilic subtypes - evaluate the eligibility for PRRT in I-refractory metastatic DTC
**EANM practice guideline for PET/CT imaging in medullary thyroid carcinoma** [8] SSTR imaging - for detection of persistent or recurrent MTC - for staging or restaging - to assess SSTR expression to select MTC patients for SSTR directed therapies - for response assessment of MTC patients to PRRT, however, the prognostic value is unclear so far
**DGN Somatostatin receptor PET/CT (SSTR-PET/CT)** [40] In principle, SSTR-PET/CT is a feasible imaging modality for patients with thyroid carcinoma.
**Thyroid cancer: ESMO Clinical Practice Guidelines for diagnosis**,** treatment and follow-up** [41] SSTR imaging - is not recommended for staging due to its relatively insensitivity - can assess the SSTR expression for evaluation of PRRT
**NCCN Clinical Practice Guidelines in Oncology** [42] SSTR-imaging may be indicated - for asymptomatic patients and detectable biomarkers - in patients in whom imaging fails to detect tumor lesions - depending on calcitonin and CEA doubling time

SNMMI: Society of Nuclear Medicine and Molecular Imaging, EANM: European Association of Nuclear Medicine, DGN: Deutsche Gesellschaft für Nuklearmedizin (German Society of Nuclear Medicine), ESMO: European Society for Medical Oncology, NCCN: National Comprehensive Cancer Network

The main indication for SSTR-based imaging in DTC is patient selection for PRRT in advanced, radioiodine-refractory disease stages. Although applied since at least 1999 in DTC, the evidence for PRRT efficacy in this context also remains limited and is mostly based on small retrospective case series with mixed response results [31, 32]. The patients included in our analysis received PRRT with [^177^Lu]Lu-DOTA-TATE according to the regulations of the German Medicinal Products Act § 13(2b) based on recommendations of the interdisciplinary tumor conferences, at a time in which newer targeted therapies based on the molecular(genetic) profile of the tumor were not yet available in all cases. It is uncertain in how far the PRRT series’ outcome is of significance in view of today’s broader therapeutic landscape, but a significant potential advantage could be a lower side effect profile of PRRT [33]. For instance, the oncocytic carcinoma patient presented in Fig. 4 would have been exposed to the risk of severe fistula formation if treated with tyrosine kinase inhibitors (TKIs) [34]. More generally, PRRT evaluation in DTC may be especially suitable for patients with advanced disease stage not (yet) eligible for TKI administration. Still, patients in our series received up to 8 cycles of [^177^Lu]Lu-DOTA-TATE in clinical routine in lack of appropriate therapeutic alternatives, implying general feasibility and tolerability of the approach in the patients included: As indicated by the results, [^18^F]SiTATE PET/CT exhibited robust radioligand uptake in metastases of DTC in these cases (even outperforming MTC), thus proving to be a highly effective tool for assessing target expression in line with the theranostic principle. Of the patients who received [^18^F]SiTATE to assess PRRT eligibility, none showed evidence of SSTR-negative disease on PET/CT. An overview of the role of SSTR imaging in diagnostic work-up of DTC patients as stated in current guidelines is given in Table 3.

The reading of [^18^F]SiTATE scans in clinical routine or in the context of clinical trials may be standardized using a radiological report system, as recently proposed [35]. However, more data and validation studies are needed to confirm reproducibility of such a system and to assess a potential clinical benefit of the latter’s use. Beyond the use of novel ^18^F-labeled SSTR-targeted PET radioligands including [^18^F]SiTATE and [^18^F]AlF-NOTA-octreotide [27], further innovative PET tracers for advanced TC are underway, e.g. Fibroblast activation protein inhibitors that may also be applied in a theranostic setting [36].

A major limitation of our analysis is the small number of patients included, which however is inherent in the peculiar combination of the imaging modality (SSTR-based PET) and the tumor entity (thyroid carcinoma). To our knowledge, the cohort of thyroid cancer patients investigated in this analysis represents the largest group thus far to undergo [^18^F]SiTATE PET/CT imaging. Different histological types of thyroid carcinoma were investigated. Despite the topological coherence and thematic link of FTC, PTC, and MTC, one must keep in mind that these entities as well as the oncocytic carcinoma case included are all distinct thyroid carcinoma entities with different histological origin and genetic background [37]. We provide the first feasibility study for [^18^F]SiTATE PET/CT in thyroid carcinoma; future studies may add a correlation of pathological markers to [^18^F]SiTATE autoradiography including binding affinity studies, as already shown in NET [38]. [^18^F]SiTATE and [^68^Ga]Ga-DOTA-TATE PET/CT images presented in this study were taken approximately 8 months apart and therefore do not allow for a typical head-to-head comparison, as also clearly stated in the results section. Therefore, additional studies are warranted to compare [^18^F]SiTATE with ^68^Ga-based tracers such as [^68^Ga]Ga-DOTA-TATE. This is also true for the comparison with other established types of PET tracers such as [^18^F]FDG.

In summary, this study is the first investigation into the use of the novel PET radioligand [^18^F]SiTATE specifically in patients diagnosed with thyroid cancer. Our data demonstrate high feasibility of [^18^F]SiTATE PET/CT in this cohort of patients with DTC and MTC. The use of [^18^F]SiTATE may overcome logistical disadvantages of ^68^Ga-based tracers and facilitate SSTR-targeted PET/CT imaging of thyroid cancer, providing a therapeutic option in a theranostic setting.

## Data Availability

The datasets used and/or analyzed during the current study are available from the corresponding author on reasonable request.
